# Complete genome sequence of *Idiomarina* sp. PL1-037 isolated from the pink hypersaline Pearse Lakes, Rottnest Island, Western Australia

**DOI:** 10.1128/mra.00157-24

**Published:** 2024-07-05

**Authors:** Crystal E. Young, Hussain Alattas, Colin Scott, Daniel V. Murphy, Ravi Tiwari, Wayne G. Reeve

**Affiliations:** 1 Bioplastic Innovation Hub, Food Futures Institute, Murdoch University, Murdoch, Western Australia, Australia; 2 School of Medical, Molecular and Forensic Sciences, Murdoch University, Murdoch, Western Australia, Australia; 3 CSIRO Advanced Engineering Biology Future Science Platform, Black Mountain Science and Innovation Park, Canberra, Australia; SUNY College of Environmental Science and Forestry, Syracuse, New York, USA

**Keywords:** halophiles, extremophiles, environmental microbiology

## Abstract

*Idiomarina* sp. PL1-037 was isolated from Pearse Lakes, Rottnest Island, Western Australia. The sequenced completed genome for PL1-037 is composed of a single chromosome (2,804,934  bp) with a GC content of 47.1%. Isolation of *Idiomarina* sp. PL1-037 provides insights about culturable extremophiles from the Pearse lakes microbiome.

## ANNOUNCEMENT

*Idiomarina* are gram-negative, rod-shaped, euryhalophiles found in a range of hypersaline environments (1). Rottnest Island (~30.4 km offshore of Perth, Western Australia) hosts athalassic soda hypersaline lakes, including Pearse Lakes (2). Here, we report the complete genome sequence of a *Idiomarina* sp. PL1-037, isolated from Pearse Lakes in May 2022, when the lakes had a pH of 8.0 ± 0.01 and salinity of 20.1% ± 1.44%.

**Fig 1:**
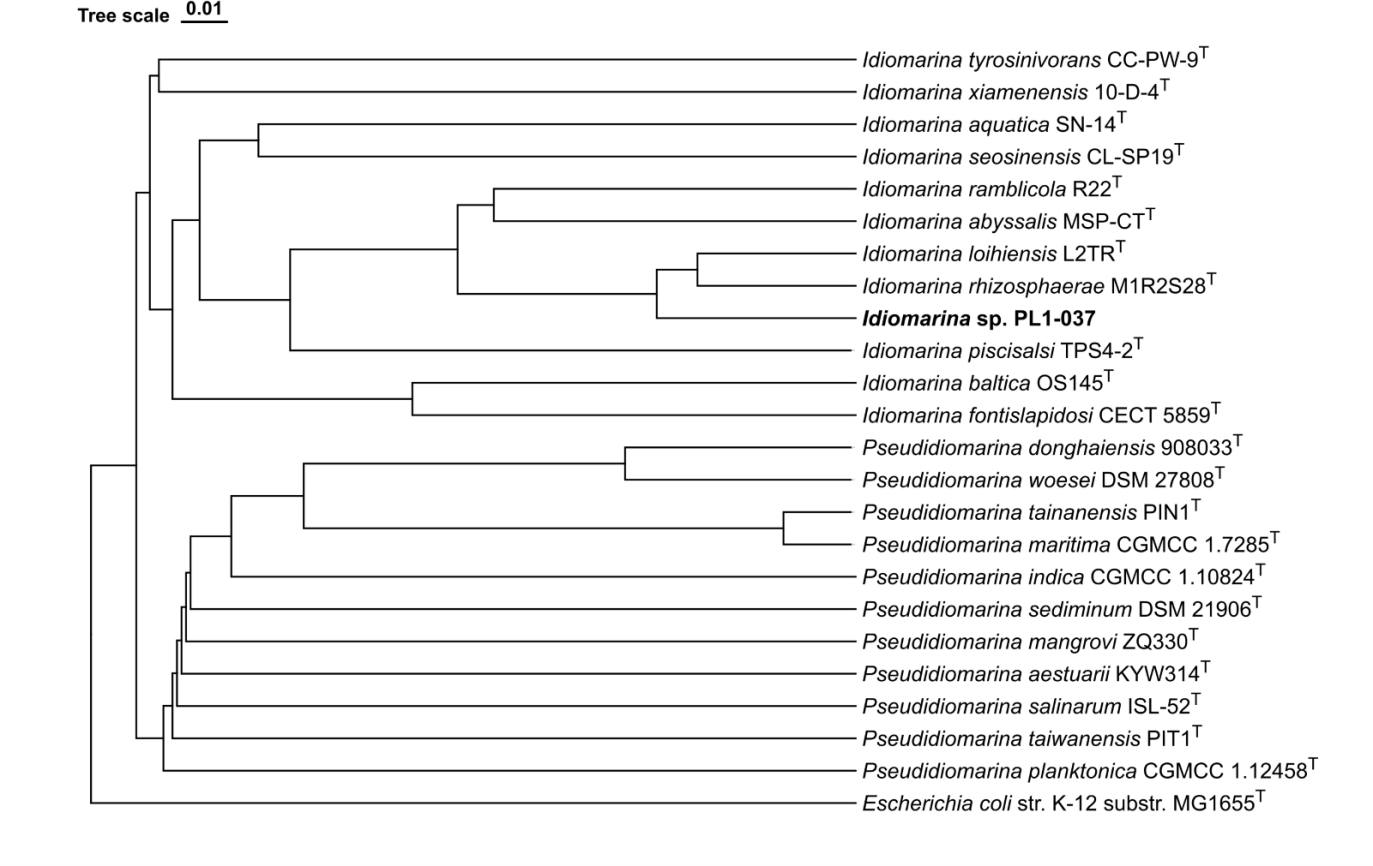
Dendro-unweighted pair group method with arithmetic mean (UPGMA) tree displaying the relatedness of *Idiomarina* sp. PL1-037 to related species based on ANIb values. The ANIb values were generated using JSpeciesWS (10) and imported into DendroUPGMA (13), and the tree was constructed using a similarity matrix (Pearson’s correlation coefficient) within the algorithm (14). The tree produced was exported in Newick format and imported into TvBOT ( https://www.chiplot.online/tvbot.html) (15). The superscript T indicates type strains, and *Escherichia coli* str. K-12 substr. MG1655 K-12 was used as an outgroup (1).

Water samples were collected (S 32° 0′ 22.281″E 115° 30′ 44.484″) and stored at 4°C before cultivation. Water samples (1,500 mL) were centrifuged at 4,500 *g* for 10 minutes and streaked from the cell pellet on modified lysogeny media containing (per liter): 10.0 g bacto-tryptone, 5.0 g bacto-yeast extract, 150.0 g NaCl, 15.0 g Agar, and 2.4 g HEPES (pH 7.8) (3). Single colonies were re-streaked until pure cultures were obtained and cryopreserved (15% glycerol, −80°C). Genomic DNA (gDNA) was extracted from stationary phase culture using CTAB (2%) (4) and sequenced using Oxford Nanopore Technology (ONT). ONT library was prepared according to the ONT ligation native barcoding gDNA library protocol (SQK-NBD114.24) (https://community.nanoporetech.com/docs) with a FLO-PRO114M flow cell (R10.4.1) on the PromethION 2 platform. Guppy [v6.5.7; (5)] was used for base calling-sequenced data with a read-pass-filter quality score cutoff value of 9 and minimum length of 1,500 bp. A total of 24,613 reads were generated (175,120,911 bp), providing an average coverage of 61× and an *N*_50_ value of 16,747 bp determined with NanoStat [v1.6.0; (6)]. ONT long reads were assembled using the Flye [v2.9.2; (7)] using default parameters with nine iterations, resulting in a single circular chromosome (2,804,934 bp) with a GC content of 47.1%. A quantitative genome assembly assessment was performed using BUSCO [v5.4.6; (8)] and CheckM [v1.2.2; (9)], providing a completeness score of 96.8% and 97.93% (0.79% contamination), respectively. Average nucleotide identity analysis using BLAST (ANIb) showed below to cut-off value (>95%) with the closest relative (91.7%) *Idiomarina rhizosphaerae* M1R2S28^T^ (GCF_024159085.1) (10 – 12) (Fig. 1).

Gene calling and annotation of the generated sequence were performed using the NCBI Prokaryotic Genome Annotation Pipeline (Table 1) [v6.6; (16)]. Isolating *Idiomarina* sp. PL1-037 and determining its complete genome will help advance our understanding of microbial life in Pearse Lakes’ extreme environment (17).

**TABLE 1:** General feature of the genome of *Idiomarina* sp. PL1-037 (accession: CP139873) from Prokaryotic Genome Annotation Pipeline

	Data from:
Feature	GenBank annotation
Total no. of genes	2,634
No. of protein-coding sequences	2,551
No. of rRNA operons	4
5S	4
16S	4
23S	4
No. of tRNA genes	56
No. of other RNA genes	4
Locus tag prefix	U0358_

## Data Availability

*Idiomarina* sp. PL1-037 whole-genome sequence (CP139873) has been deposited in GenBank database (NCBI) under the BioSample: SAMN38237614 within BioProject: PRJNA1039979. ONT long reads were deposited in SRA (SRR26974595).
